# A review of infant growth and psychomotor developmental outcomes after intrauterine exposure to preeclampsia

**DOI:** 10.1186/s12887-022-03542-5

**Published:** 2022-08-30

**Authors:** Priya Vakil, Amanda Henry, Maria E. Craig, Megan L. Gow

**Affiliations:** 1 School of Women’s and Children’s Health, UNSW Medicine, Sydney, Australia; 2 Department of Women’s and Children’s Health, St George Hospital, Sydney, Australia; 3 The George Institute for Global Health, Sydney, Australia; 4 University of Sydney Children’s Hospital Westmead Clinical School, Sydney, Australia

**Keywords:** Preeclampsia, Hypertensive pregnancy, Infant, Growth, Development

## Abstract

Preeclampsia is a hypertensive disorder of pregnancy with serious health implications for mother and their offspring. The uteroplacental vascular insufficiency caused by preeclampsia is associated with epigenetic and pathological changes in the mother and fetus. However, the impact of preeclampsia in infancy (birth to 2 years), a time of rapid development influenced by pre- and postnatal factors that can predict future health outcomes, remains inconclusive. This narrative review of 23 epidemiological and basic science studies assessed the measurement and impact of preeclampsia exposure on infant growth and psychomotor developmental outcomes from birth to 2 years. Studies assessing infant growth report that preeclampsia-exposed infants have lower weight, length and BMI at 2 years than their normotensive controls, or that they instead experience accelerated weight gain to catch up in growth by 2 years, which may have long-term implications for their cardiometabolic health. In contrast, clear discrepancies remain as to whether preeclampsia exposure impairs infant motor and cognitive development, or instead has no impact. It is additionally unknown whether any impacts of preeclampsia are independent of confounders including shared genetic factors that predispose to both preeclampsia and childhood morbidity, perinatal factors including small for gestational age or preterm birth and their sequelae, and postnatal environmental factors such childhood nutrition. Further research is required to account for these variables in larger cohorts born at term, to help elucidate the independent pathophysiological impact of this clinically heterogenous and dangerous disease.

## Introduction

Approximately 3–5% of women worldwide experience preeclampsia, a multisystem hypertensive disorder of pregnancy (Table 1) [1–3]. Preeclampsia represents a significant maternal health burden with complications including perinatal mortality and increased lifetime risks of cardiometabolic diseases such as hypertension, stroke, ischaemic heart disease and type 2 diabetes mellitus [2, 4–9].

**Table 1:** Hypertensive disorders of pregnancy: definitions and associated features

Hypertensive Disorder	Definition of hypertension	Associated Features
Chronic hypertension	Onset before pregnancy or before 20 weeks’ gestation: − ≥ 140 mmHg SBP or − ≥ 90 mmHg DBP	− Mainly due to essential hypertension − 24-h ambulatory BP monitoring assists the exclusion of white-coat hypertension − Risk factor for preeclampsia, maternal CVD and FGR
Gestational hypertension	New onset at or after 20 weeks’ gestation: − ≥ 140 mmHg SBP or − ≥ 90 mmHg DBP	− May be transient in nature, arising and settling in the 2^nd^-3^rd^ trimester − 25% will progress to preeclampsia − Return to normal BP postpartum with no antenatal proteinuria or maternal end-organ dysfunction − Increased future risk of maternal CVD
Preeclampsia	New onset at or after 20 weeks’ gestation with end-organ dysfunction: − ≥ 140 mm Hg SBP or − ≥ 90 mm Hg DBP	New onset of ≥ 1: − Proteinuria − Acute Kidney Injury − Elevated liver transaminases − Neurological complications − Thrombocytopenia − Uteroplacental dysfunction − FGR − HELLP syndrome (haemolysis, elevated liver enzymes, thrombocytopaenia)
Eclampsia	New onset of antenatal, intrapartum or postpartum tonic–clonic, focal, or multifocal seizures without other causative conditions	Often preceded by: − Severe and persistent occipital or frontal headaches − Blurred vision − Photophobia − Altered mental status

Abbreviations: *BP* Blood pressure, *CVD* Cardiovascular disease, *DBP* Diastolic blood pressure, *FGR* Fetal growth restriction, *mmHg* millimetres of mercury, *SBP* Systolic blood pressure

In preeclampsia, pathological mechanisms such as uteroplacental vascular insufficiency create an unfavourable intrauterine environment [10, 11], which lead to many extensively studied fetal and neonatal complications [2, 7, 10–15]. In children and adults, intrauterine preeclampsia exposure is associated with an increased risk of cardiovascular, metabolic, immune, respiratory, and neurodevelopmental morbidities [10, 16–21]. One explanation is the Developmental Origins of Health and Disease (DOHaD) hypothesis, which suggests that the fetal adaptation to the adverse intrauterine environment increases future chronic disease risk [10]. Alternatively, others suggest that shared genetic or environmental risk factors predispose to future maternal and paediatric morbidity [16, 22].

There is some evidence for impaired growth and psychomotor neurodevelopment in infancy (birth to 2 years) after preeclampsia exposure [23, 24], but much of the existing data are limited by their minimal adjustment for perinatal confounders, the variable use of assessment tools for growth and development, and their specific study cohorts of preterm or very low birthweight (VLBW) infants (Table 1 and 2). Robust early detection of abnormal growth and development trajectories may aid the development of novel therapeutic interventions to improve childhood health outcomes for infants exposed to preeclampsia. We aimed to determine whether infants with intrauterine preeclampsia exposure, compared to infants born from normotensive pregnancies, have differing anthropometric growth outcomes and psychomotor developmental outcomes from birth to 2 years of age. Thus, we review the fetal, neonatal and long-term consequences of preeclampsia exposure, discuss differing ways to measure infant growth and developmental outcomes, and review studies of infant growth and psychomotor development associated with preeclampsia exposure.

**Table 2:** Studies assessing the impact of preeclampsia exposure on growth in infancy (birth – 2 years)

**First Author (Year)**	**Study Type**	**Exposure (Number)**	**Outcomes Assessed**	**Main Findings at 2 Years**^a^ **(PE versus NTP-exposed infants)**	**Adjusted Confounders**	**Comments**
Szymonowicz (1987) [23]	Prospective case–control	PE (35) NTP (35)	Weight	PE lower	Nil	Cohort: preterm, VLBW infants^b^ ROB: Low
			Length	ND		
			Head circumference			
Martikainen (1989) [24]	Prospective cohort	PE (31 preterm, 40 term) NTP (128 preterm, 175 term)	Weight Length	Preterm: PE lower Term: ND	Infant sex, GA	Also assessed other HDPs Cohort stratified by hypertension exposure, prematurity and SGA status. Assessed 18-month outcomes ROB: Low
			Weight gain Length gain	Term PE: greater catch up than preterm PE infants		
			Head circumference	Preterm: ND Term: PE higher		
Cheng (2004) [25]	Retrospective cohort	PE (28) NTP (61)	Weight	ND	Nil	Cohort: very preterm (< 32 weeks), VLBW infants^b^. Small sample size ROB: Low
			Length			
			Head circumference			
Silveira (2007) [26]	Prospective cohort	PE (40) NTP (46)	Weight, Weight-for-age	PE lower, slower catch-up weight in VLBW PE than VLBW NTP	GA	Cohort: preterm, VLBW infants^b^. Assessed 12, 18-month outcomes ROB: Low
			Length-for-age	ND		
			Head circumference	PE lower		
			Weight-for-length			
Davis (2015) [18]	Prospective cohort	C-HTN^c^ (89) NTP (1434)	Weight	ND	Infant sex, GA, birthweight	Grouped PE and GH causing preterm birth into C-HTN Assessed 12-month outcomes. Assessed growth and CVD risk to 20 years ROB: Low
			Length			
			BMI			
Byberg (2017) [27]	Nested case–control	S-PE^c^ (54) M/M-PE^c^ (164) NTP (385)	Weight z-score	PE lower (all)	Infant sex, age Maternal age, BMI, antenatal smoking, education	Considered severity of PE Assessed growth to 13 years No adjustment for GA or birthweight ROB: Low
			Length z-score gain	M/M-PE boys greater, S-PE boys and all girls lower		
			BMI	MM-PE girls greater, S-PE girls and all boys lower		
Matić (2017) [28]	Retrospective cohort	GH/PE (261) NTP (1212)	Weight	GH/PE lower	Nil	Grouped PE and GH Cohort: 2–3 year old infants born very preterm (< 29 weeks)^b^. Powered for chronic lung disease and neurodevelopment ROB: Low
			Length			
			Head circumference			
Gunnarsdottir (2018) [29]	Retrospective cohort	S-PE^c^ + M/M-PE^c^ (865) NTP (22,898)	Length z-score	S-PE lower	Infant sex, GA, birthweight, SGA status, breastfeeding status Maternal age, parity, height, BMI, diabetes, smoking, education, country of birth Paternal smoking	Assessed 18-month outcomes. Assessed growth from birth to 5 years. No adjustment for paternal factors influencing length ROB: Low
			Length gain	All PE greater, especially S-PE than M/M-PE infants, partly associated with GA		
Huang (2020) [30]	Prospective cohort	PE (24) NTP (168)	BMI	PE greater	Infant sex, GA, birthweight Maternal age, parity, gestational diabetes mellitus, education, marital status	Considered association of both gestational diabetes mellitus and PE on growth. Assessed 18, 24-month outcomes. Assessed growth to 6 years. Small sample size ROB: Low
Gow (2021) [31]	Prospective cohort	PE (84) NTP (298)	Weight Weight z-score	PE lower	Infant sex, GA, NICU/SCN stay length, feeding status, labour onset, mode of delivery Maternal age, weight, BMI, parity, ethnicity, smoking, education	Assessed 6-month outcomes ROB: Low
			Weight gain	PE greater		
			Weight z-score gain Rapid weight gain Conditional weight gain	ND, any SGA greater than not SGA		
			Length Length z-score	PE lower ND		
			Length gain Length z-score gain	PE greater ND		
			BMI	ND		
Jasper (2021) [32]	Retrospective cohort	PE (659) NTP (1909)	Rate of weight z-score gain	ND	Infant birthweight, GA, head circumference, multiple birth, postnatal hospitalisation, year of birth, mode of delivery, perinatal complications Maternal age, BMI, ethnicity, SES	Cohort: preterm infants^b^ Many perinatal exposures assessed, including PE ROB: Low

*Abbreviations*: *BMI* Body mass index, *C-HTN*^c^ Complicated hypertension exposed, *CVD* Cardiovascular disease, *FGR* Fetal growth restriction, *GA* Gestational age at birth, *GH* Gestational hypertension, *M/M-PE*^c^ Mild/moderate preeclampsia, *ND* No difference, *NICU/SCN* Neonatal intensive care unit/special care nursery, *NTP* Normotensive pregnancy, *PE* Preeclampsia, *ROB* Risk of bias, *SGA* Small for gestational age, *S-PE*^c^ Severe preeclampsia, *VLBW* Very low birth weight

^a^All results in the ‘Main Findings’ column are of infant growth outcomes at 2 years, unless specified in the ‘Comments’ column. Any study that continued reporting outcomes beyond 2 years is also specified

^b^Preterm birth was defined as birth < 37 weeks’ gestation. VLBW was defined as birthweight < 1500 g. SGA birth was defined as birthweight corrected for gestational age < 10^th^ centile. Study-specific definitions of ‘very preterm’ are specified in the ‘Comments’ column

^c^Mild/moderate PE and severe PE definitions differed between studies: Davis et al. (2015) [18] combined PE and gestational hypertension severe enough to result in preterm delivery into C-HTN; Byberg et al. (2017) [27] used criteria developed by the CLASP study based on blood pressure and proteinuria levels at GA 20 weeks [33, 34]; Gunnarsdottir et al. (2018) [29] used the WHO ICD-10 classifications [35]

## Methods

We searched PubMed, Medline and Embase using search terms: preeclampsia AND (infant OR child) AND (growth OR weight OR length OR development OR neurodevelopment), Google Scholar with key words preeclampsia, hypertensive disorders of pregnancy, child, infant, growth, development, neurodevelopment, health, and the gray literature to identify cohort or case–control studies, published any date to 31^st^ October, 2021, without language restriction or full-text restriction, that assessed infant growth or development after preeclampsia exposure.

Inclusion criteria were outcome data on infant growth (weight, length, BMI, weight for age, weight for length, growth trajectories and other anthropometric measures) and psychomotor neurodevelopment (gross and fine motor, expressive and receptive communication, social, personal and cognitive skills) from birth up to and including 2 years of age in infants with intrauterine preeclampsia exposure. Studies were also included if other hypertensive disorders of pregnancy such as gestational hypertension, were combined with preeclampsia or included as a separate exposure group in addition to preeclampsia exposure, or if preeclampsia exposure was stratified according to severity or timing of onset, for example, in the case that no normotensive group was compared. Studies were excluded if they only reported birth outcomes, did not include outcomes reported between birth to 2 years of age, but were included if they reported later outcomes in addition to this age range. Studies were also excluded if data were reported on other hypertensive disorders not including preeclampsia.

Eligible studies were critically appraised by two reviewers (PV and MLG) for methodological quality using the Joanna Briggs Institute (JBI) Critical Appraisal Checklist for Cohort Studies and Case–Control Studies, with possible answers including “yes”, “no”, “unclear” or “not applicable” [36]. After discussion and agreeance between reviewers about cut-off values as suggested in the JBI Manual for Evidence Synthesis, the studies were categorised as either of low risk (≥ 70% “yes”), moderate risk (50–69% “yes”), or high risk (< 50% “yes”) of bias [36, 37].

## Background

### Intrauterine complications of preeclampsia and the DOHaD hypothesis

Barker et al. [38–41] were the first to suggest that a chronic, non-communicable disease in adulthood- ischaemic heart disease, was associated with exposure to an intrauterine environment that inhibited fetal growth and nutrition. Barker’s hypothesis was extended by studies that controlled for confounders including gestational age at birth, genetic risk factors and postnatal environmental factors [19, 42]. They found independent associations between fetal growth restriction (FGR) and a wider range of chronic diseases, resulting in the DOHaD hypothesis.

**Fig. 1:**
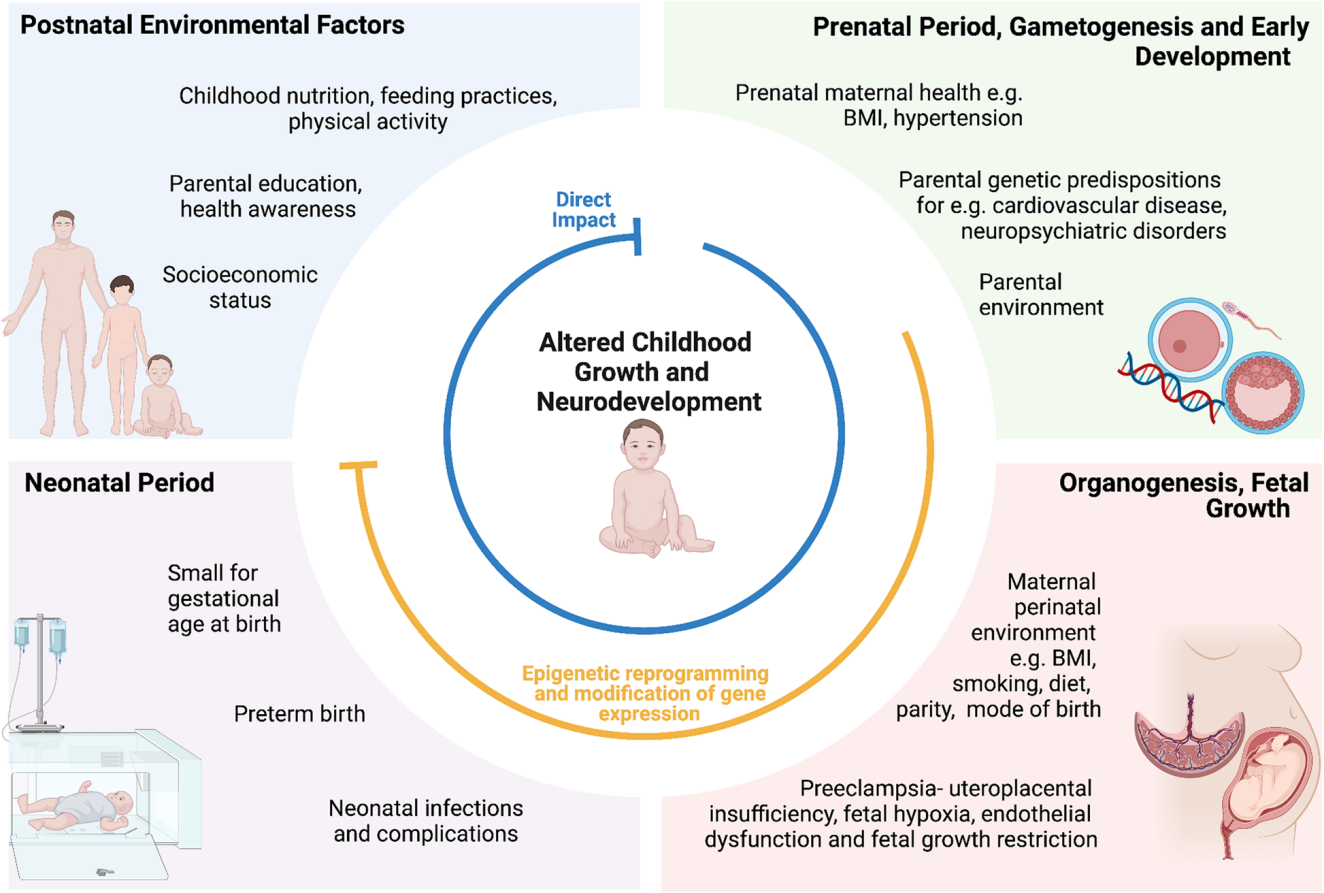
Factors associated with preeclampsia and mechanisms by which they may impact childhood growth and neurodevelopmental outcomes

Epigenetics refers to phenotype changes caused by alterations in gene expression rather than hereditary changes in the DNA sequence itself. Epigenetic changes occur in both developing and differentiated tissue through mechanisms including DNA methylation, histone modification and the action of micro- and noncoding-RNAs [43–45]. These mechanisms can be influenced by the perinatal maternal, paternal and postnatal environment, and in line with the DOHaD hypothesis, may impact the offspring’s future health [45]. Although the pathogenesis of preeclampsia is incompletely understood, proposed mechanisms include immunological imbalances, pre-existing comorbidities including obesity and chronic hypertension, and epigenetic changes in the placenta and maternal circulation, which lead to defective placentation and incomplete trophoblast invasion into the myometrial spiral arteries in early pregnancy. Subsequent angiogenic imbalances, placental hypoperfusion and ischaemia, and systemic maternal inflammation and oxidative stress occur, with associated fetal endothelial dysfunction, hypoxia and malnutrition of varying severity [11, 43, 44]. It is hypothesised that the fetus undergoes ‘developmental programming’ as an adaptation to this adverse intrauterine environment, which may increase their future risk of morbidity [43–45] (Fig. 1).

The DOHaD hypothesis suggests that greater intrauterine preeclampsia-exposure, irrespective of shared genetic or lifestyle factors, has a programming effect that impacts the child’s development of morbidities. For example, a large population-based cohort study (*n* = 758,524) [46] demonstrated a higher relative risk of long-term morbidity in offspring the longer the intrauterine preeclampsia-exposure. However, the investigators were unable to control for maternal body mass index (BMI), smoking, lifestyle factors or diet, all possible contributors to childhood morbidity. In sibling studies, children exposed to preeclampsia had increased vascular dysfunction [19] and higher risks of developing neurodevelopmental morbidities [47] than their unexposed sibling, supporting an intrauterine programming effect of preeclampsia.

Conversely, others suggest that shared genetic or environmental risk factors that predispose to future paediatric morbidity, or even preeclampsia itself such as maternal cardiometabolic disease, are responsible for the increased disease risk observed in exposed children [16, 22] (Fig. 1). This may also explain why late-onset preeclampsia with uteroplacental disease of differing severity, or gestational hypertension, which does not typically demonstrate the intrauterine complications of preeclampsia, are also associated with increased risks of childhood morbidity [3, 48–50]. 

Figure 1: In the prenatal, perinatal, and postnatal periods, factors associated with preeclampsia such as genetic risk factors shared between parent and child, intrauterine changes, and external environmental influences including neonatal complications, parent health behaviours and the postnatal lifestyle, may contribute to altered childhood health outcomes. These factors can directly influence childhood growth and development, or may induce epigenetic reprogramming during fetal and neonatal development that can subsequently increase child future chronic disease risk. Created with BioRender.com

### Perinatal and neonatal outcomes after preeclampsia exposure

Preeclampsia is associated with adverse fetal outcomes including FGR, placental abruption, stillbirth, and neonatal mortality [7, 51]. Approximately 12–33% of preeclampsia-exposed neonates are born small for gestational age (SGA, birthweight z-score corrected for sex and gestational age < 10^th^ centile) [52–55]. Delivery is the only definitive management of preeclampsia to prevent progression to end-organ damage [56]. Subsequently, many neonates are born premature, with associated sequelae including nursery admission, respiratory distress syndrome, bronchopulmonary dysplasia, and sepsis [7, 12, 57].

Clinically, preeclampsia is a heterogenous disorder with poorer perinatal outcomes generally associated with early-onset (< 34 weeks’ gestation) and preterm (34 – 36 + 6 weeks’ gestation) preeclampsia compared to that diagnosed at term (≥ 37 weeks). This may be due to the complications of preterm delivery, or longer fetal exposure to the adverse intrauterine environment, resulting from the greater uteroplacental dysfunction in early-onset (versus term) preeclampsia [13, 49, 50, 58]. Nonetheless, the intrauterine and perinatal complications of preeclampsia are associated with adverse paediatric outcomes [59].

### Long-term paediatric outcomes after preeclampsia exposure

Preeclampsia exposure has been associated with increased long-term paediatric cardiometabolic risk, including increased blood pressure [16, 60–64] and BMI [16, 17, 63], altered cardiac structure [65] and vascular function [19, 66], and increased stroke [67] and hypertension risk [21, 67]. While preeclampsia exposure has also been associated with increased risks of neurodevelopmental disorders including autism spectrum disorder [68–71], attention deficit hyperactivity disorder [72–74], epilepsy [75, 76], impaired motor development [77, 78], mild cognitive impairment or neurodevelopmental delay [79–83], cerebral palsy [84–86] and mood disorder symptoms [87], some studies suggest it has a neuroprotective effect [88]. Furthermore, preeclampsia is linked to immunological impairment in exposed offspring, including increased risk of asthma and other respiratory diseases [20, 75, 89, 90], atopy and allergic sensitisation [90–92], and allergic rhinoconjunctivitis [92].

The strengths of these studies are that most had relatively large sample sizes and adjusted for putative genetic and lifestyle confounders, including maternal demographic variables like BMI, prior comorbidities and ethnicity, and neonatal factors including prematurity status, gestational age, SGA status, and special care nursery stay [7, 10, 59, 93]. However, heterogenous findings between studies could be explained by the nonstandard adjustment of these potential confounders, and further replication of results is needed for lesser studied morbidities such as stroke [67] and allergic rhinoconjunctivitis [92]. Furthermore, few studies adjusted for confounding lifestyle factors such as childhood nutrition [10] which influence cardiometabolic health, and despite adjustment for maternal CVD, the genetic inheritability of chronic morbidities like CVD are difficult to exclude. Furthermore, few studies considered preeclampsia severity or onset, which, given the clinically heterogeneity of preeclampsia, may significantly alter paediatric outcomes [93]. Hence, while the longer-term paediatric consequences of preeclampsia have been investigated, more targeted research is needed to validate and replicate current findings, and disentangle the impact of genetic and lifestyle factors from preeclampsia exposure itself.

## Infant growth after preeclampsia exposure

Growth in infancy (birth – 2 years) is rapid, non-linear, and a key indicator of health and nutritional status. Infant growth is influenced by many factors including genetics, feeding patterns, nutrient composition, metabolic and hormonal signals, environmental influences and underlying pathological processes [94–96]. Rapid growth in infancy can reflect underlying genetic, cardiovascular, metabolic, endocrine, or gastrointestinal morbidities including preeclampsia exposure, and is associated with increased future risks of obesity, metabolic syndrome, and CVD. Poor growth in infancy may indicate poor nutritional status, underlying genetic conditions or morbidity associated with FGR such as that experienced in preeclampsia, and is associated with later neurological, cardiovascular, renal, and respiratory morbidity [94–98]. Elucidating the impact of preeclampsia on growth is hence of utmost clinical significance.

### Assessment of infant body composition

Body composition assessments, including anthropometric measurements of weight, length, head, abdominal and mid-upper arm circumferences, and triceps and subscapular skinfold thicknesses act as clinical screening tools to monitor infant growth and risk of future morbidity [99]. Body proportion metrics derived from height and weight measures include weight-for-length and BMI. Weight-for-length is currently recommended by the World Health Organisation (WHO) and has been adopted internationally to assess body proportionality in infants aged ≤ 2 years [100–102]. It considers the positive relationship between height and weight and is a useful indicator of nutritional status when infant age is unknown, however it is not adjusted for age-dependent variations and is a suboptimal indicator of adiposity [103–105]. In contrast, BMI (weight in kilograms/ height in metres squared) has a higher correlation with fat mass, fat-free mass and percent body fat z-scores than weight-for-length. It is also adjustable for infant age, including gestational age, to assess infant growth over time [104–106]. Although the ponderal index (weight in grams × 100/ length in centimetres cubed) has been considered a more appropriate measure of proportional growth in preterm infants in the past, BMI may have a stronger correlation with fat measures and is also a suitable measure of preterm infant body proportionality [107]. Considering BMI z-scores are currently recommended for assessing growth in children older than 2 years, measuring BMI in infancy may also provide a more consistent growth assessment in primary care settings [104–106]. However, one large prospective cohort study found the choice of weight-for-length compared to BMI z-scores did not greatly affect the association with future cardiometabolic outcomes, suggesting either are suitable measures of infant growth [106].

### Assessment of longitudinal infant growth

The WHO Child Growth Standards charts are validated standards to calculate an infant’s age- and sex-adjusted growth relative to the population mean [100, 101]. The Fenton Preterm Growth Charts, revised in 2013, are established standards developed to assess the size of preterm infants at birth [108]. However, they do not consider the postnatal physiological weight loss experienced by infants in the first days of life, and thus are unsuitable for assessing the longitudinal growth of preterm infants [109]. The INTERGROWTH-21(st) Preterm Postnatal Growth Standards [110] may be more accurate for preterm populations as they consider the differing postnatal growth patterns in the first 6 months that preterm neonates experience. They were developed from the postnatal growth of preterm infants born without morbidity from uncomplicated pregnancies across 8 countries, and have high concordance with the Fenton Preterm Growth Charts, identifying slightly greater numbers of SGA infants at birth. Importantly, these additional infants identified had higher incidences of morbidity than those identified by the Fenton Charts, supporting the use of the INTERGROWTH-21(st) Standards in preterm populations [111, 112]. However, they were developed from only 201 infants and require further international validation in larger, ethnically and socioeconomically diverse populations. As the postnatal growth of preterm infants converges with term infants by 6 months, the WHO standards are appropriate for all infants 6 months onwards [110].

Weight-for-age z-scores assess longitudinal infant growth, and BMI or weight-for-length z-scores assess proportionality change [101]. Rapid weight gain is defined as a > 0.67 gain in weight-for-age z-score, corresponding to crossing two centile lines on respective growth charts, and is associated with future CVD risk [113]. Infants who suffered FGR and were subsequently born SGA, a common complication of preeclampsia, often experience necessary rapid weight gain as a recovery response to intrauterine undernutrition [114]. This is referred to as ‘rapid catch-up growth’; an example of how infants born on weight extremes may experience natural regression to the mean postpartum [115], and also how infant weight may vary dynamically relative to weight-for-age growth curves [113]. Current interpretations of WHO weight-for-age curves assume children may normally not deviate from their initial weight standard deviation (SD) score [101], and thus weight-for-age changes can represent pathological growth trajectories in otherwise healthy children.

For infants 0–6 months, this limitation of weight-for-age z-scores may be overcome using conditional weight gain z-scores. This compares current infant weight with that predicted from their previous weight to derive a weight gain SD score, and references this to a conditional reference which considers the tendency of infants on the extremes of weight to experience non-pathological regression to the mean [31, 115–117]. For infants 6–24 months, including those born premature or SGA, BMI or weight-for-length z-scores are alternative metrics to assess growth that may account for the limitations of weight-for-age z-scores [118].

### Results: growth outcomes of infants exposed to preeclampsia

While is it well established that preeclampsia is associated with FGR and both premature and SGA birth, it is still unclear whether preeclampsia has an intrauterine programming effect impacting infant growth trajectories independent of these perinatal and other genetic and lifestyle confounders [15, 57, 119]. Furthermore, although all classifications of preeclampsia are considered clinically significant and potentially life-threatening for mother and child [50], early onset or more severe preeclampsia may reflect greater placental dysfunction that can impact fetal, neonatal and childhood growth differently to later onset, mild or moderate disease [49].

Our search identified 11 studies that assessed infant growth outcomes after preeclampsia exposure. All studies were assessed with the JBI tool to have a low risk of bias. (Table 2). Six of these reported that infants exposed to preeclampsia had lower weight and BMI throughout infancy, remaining smaller at multiple timepoints from birth to 2 years than infants of normotensive pregnancies [23, 24, 26–28, 31]. Two cohort studies of preterm, VLBW (< 1500 g) infants, found those exposed to preeclampsia had significantly lower absolute weight, weight z-scores and weight-for-length z-scores throughout infancy [23, 26]. In preterm infants, two studies also report an association with preeclampsia and lower weight [24, 28], however the latter study grouped preeclampsia and gestational hypertension exposure and found no difference in weight in term infants compared to those born from normotensive pregnancies. While this suggests that the impact of preeclampsia may vary across the gestational spectrum, it may instead reflect the impact of early-onset or more severe preeclampsia that is often the cause of premature birth [49, 50]. This is supported by Byberg et al. (2017), who reported lower BMI z-scores from infancy in those exposed to more severe preeclampsia [27]. However, although these studies demonstrate associations between preeclampsia and poor infant growth, they did not adjust for the confounding influence of premature or VLBW birth, which are independently associated with infant growth restriction [15, 24, 57, 119]. This limits the isolation of the specific pathophysiological implications of preeclampsia exposure independent of these confounders.

In contrast, three studies have reported no difference in weight or BMI in infants exposed to preeclampsia or normotensive pregnancies in late infancy [24, 25, 120]. Davis et al. (2015) [18] reported preeclampsia and gestational-hypertension-exposed neonates were not significantly smaller in birthweight when adjusted for gestational age and had no differences in weight z-score or BMI at 12 months compared to infants of normotensive pregnancies. However, Martikainen et al. (1989) [24] reported that preeclampsia-exposed infants who were born significantly smaller at term, similarly had no difference in weight to normotensive infants by 18 months, suggesting they had an accelerated growth trajectory that enabled ‘catch up’ growth. This may suggest a relationship of preeclampsia exposure with accelerated growth independently, or in conjunction with SGA birth that, although associated with impaired infant growth in some infants, is a cause of rapid weight gain in others as a response to intrauterine undernutrition [114, 115]. Both Gow et al. (2021) [31] and Jasper et al. (2021) [32] investigated this relationship, and while they reported associations between preeclampsia exposure and weight gain throughout infancy, preeclampsia exposure was no longer a significant contributor to this catch-up growth after full adjustment for confounders like SGA status and maternal BMI. Overall, this suggests that the pathological mechanisms of preeclampsia may have no independent impact on infant weight gain. However clinically, preeclampsia and its associated comorbidities have been associated with increased growth trajectories and rapid weight gain, leading to greater BMIs in late infancy [30], greater weight and BMI from school age onwards in females especially [27], and a threefold risk of being hypertensive by age 20 [18].

Similar discrepancies regarding infant length and length gain are present. Although Martikainen et al. (1989) [24] and Matić et al. (2017) [28] reported that in preterm infants, those exposed to preeclampsia continued to have lower lengths in late infancy, this trend did not persist for term infants, potentially reflective of the impact of more severe or early-onset PE that these preterm infants may have experienced. Five other studies reported no difference in length or length z-scores in late infancy [18, 23, 25, 26, 31], reflecting either minimal differences in length at birth between groups, or for preeclampsia-exposed infants born small, the catch-up growth they experienced. Interestingly, while Gunnarsdottir et al. (2018) [29] reported no length differences in infants exposed to mild or moderate preeclampsia versus normotensive pregnancies, those with severe preeclampsia exposure had lower length z-scores at 18 months. This supports the notion that preeclampsia may encompass pathologically diverse diseases grouped by onset or severity that impact infant growth heterogeneously. In infants exposed to severe preeclampsia, Gunnarsdottir et al. (2018) [29] additionally reported greater absolute length gain, while Gow et al. (2021) [31] reported no difference in length z-score gains, and Byberg et al. (2017) [27] lower length z-score gains. The heterogenous findings of these studies may be partially mediated by gestational age and SGA status.

Head circumference differences between infants exposed to preeclampsia versus normotensive pregnancies have also been explored [23–26, 28]. In preterm or VLBW infants, preeclampsia exposure seems to minimally contribute to differences in head circumference, or be associated with lower head circumferences throughout infancy [23–26, 28]. When considering only those born at term, Martikainen et al. (1989) [24] demonstrated that preeclampsia exposure was associated with larger head circumferences. While potentially confounded by the influence of SGA status, this finding may suggest an independent impact of preeclampsia, and support the differences between severe, earlier-onset preeclampsia more common in preterm infants, versus the moderate or later onset disease positively associated with growth in term infants.

In general, there are many discrepancies between the studies assessing the impact of preeclampsia exposure on growth. Despite demonstrating low risks of methodological bias, these studies had limitations such as differing adjustment for confounders due to a lack of collected data, specific cohorts of premature or VLBW infants, or deliberate choice to consider the intermediate relationship of the confounder with preeclampsia and growth [27]. Furthermore, lack of adjustment for postnatal infant nutrition and other environmental influences may lead to an overestimation of the impact of preeclampsia exposure. Also, certain studies had smaller sample sizes [23, 25, 30], were designed to assess multiple perinatal comorbidities rather than preeclampsia specifically [18, 30, 32], or compared differing subgroups of preeclampsia severity [18, 27, 29]. As such, the impact of intrauterine preeclampsia exposure on growth in infancy, either independent of confounders like SGA or prematurity status in line with the DOHaD hypothesis, or in conjunction with inherited genetics that predispose to both preeclampsia and cardiometabolic disease, remain uncertain. Nevertheless, preeclampsia exposure remains a clinically significant risk factor that highlights opportunities to monitor infants into later childhood, and may indicate a need for early clinical intervention.

## Infant development after preeclampsia exposure

Infant psychomotor development refers to the maturation of the brain and central nervous system in four main domains: gross and fine motor skills, speech and language, performance and cognition, and social and personal skills [121]. Despite being a dynamic process influenced by genetic, perinatal, and environmental factors, normal development generally occurs in an ordered and sequential pattern correlating to age-dependent developmental milestones [121, 122].

### Assessment of infant development

Developmental assessment is a longitudinal process involving joint surveillance by both clinicians and parents [123]. Developmental screening tools assist the identification of potential developmental delay, defined in infants as > 2SDs below the mean on age-appropriate standardised testing [124]. The Ages and Stages Questionnaire (ASQ) [125], Parents Evaluation of Developmental Status [126] and Survey of Well-being of Young Children [127] are commonly used, parent-completed screening surveys that assess many domains including fine and gross motor, receptive and expressive communication, problem solving, and personal or social skills. These tools consider parental observation which may increase their sensitivity [123, 128–130], however, they may not be suitable for infants younger than 4 months or those with special needs [125, 127, 129]. The Parent Report of Children’s Abilities-Revised [131, 132] is useful for screening preterm infants, while the parent-completed Child Development Inventory [133] can assist identification of children with special needs [129, 133]. Similarly, the child-administered Battelle Developmental Inventory Screening Tool, 2^nd^ edition [134, 135] can be modified for special needs children to assess psychomotor development. The Denver Developmental Screening Test, 2^nd^ edition [136] and the Brigance Screens [137] also assess infant psychomotor domains through direct elicitation and observation of the child, however are longer to administer (10–20 min) than parent-completed surveys [129, 137]. While these screening tools are generally simple, quick and cost effective to implement in a primary care setting, they are not diagnostic, so children identified at risk of developmental delay require specialist diagnostic developmental assessment [121, 123].

While no gold standard assessment tool exists, the Bayley Scales of Infant Development 2^nd^ (BSID-II) [138] and 3^rd^ (BSID-III) [139] editions are the most commonly used and validated psychometric assessments used in infancy for both clinical and research purposes [122, 123]. These assessments assist the identification, and for the BSID-III, quantification of developmental delay in infant psychomotor (PDI) and mental (MDI) developmental indices. While the BSID-II MDI score was additionally useful in determining cognitive function in preterm or low birthweight infants, the BSID-III may have reduced sensitivity in these populations [140], and both are long assessments which may provide more difficulty for clinician, parent and infant [122, 123, 140]. The Griffiths Mental Development Scale, 2^nd^ Edition [141] is another assessment with concurrent validity to the BSID-II that may be more successful than the BSID-II at detecting motor delays in infancy, however it may not be as sensitive for detecting speech and language delay. It also has a subsequent scale from ages 2–8, which may be useful for longitudinal childhood developmental surveillance [122, 141]. Furthermore, assessment tools like the Mullen Scales of Early Learning [142] may be useful for assessing the cognitive development of infants without or with autism spectrum disorder or known developmental delay. While many developmental assessments measure similar domains and have concurrent validity, scores are often measured on differing scales and thus clinically, should not be interchanged between tools to prevent inaccurate approximation of infant ability [122, 143].

### Results: developmental outcomes of infants exposed to preeclampsia

Although intrauterine preeclampsia exposure has been associated with impaired psychomotor development in older children, adolescents, and adults [83, 144], there are uncertainties regarding its effects on psychomotor development in infancy (birth – 2 years).

Our search identified 17 studies assessing infant psychomotor development after preeclampsia exposure. All studies were assessed with the JBI tool to have a low risk of bias. (Table 3). Most studies were conducted on specific populations of infants, such as those born preterm, of VLBW or SGA, comorbidities independently associated with poorer neurodevelopment [15, 145–147]. For example, in cohorts of preterm infants, Spinillo et al. (1994) [148] reported lower BSID mental and psychomotor developmental index scores, and Johnson et al. (2015) [149] poorer cognitive outcomes, in 2 year old infants exposed to preeclampsia after adjustment for SGA status and other covariates. Similarly, Martikainen et al. (1989) demonstrated infants exposed to preeclampsia born preterm had poorer fine motor skills and visuo-auditory perception at 18 months than normotensive controls, while term infants had better motor skills, visuo-auditory perception and social abilities. This may reflect the impact of more severe or early-onset preeclampsia, which are associated with greater uteroplacental deficiencies and are often the cause of preterm birth [49, 150]. In contrast, other studies in preterm populations reported no difference in infant neurodevelopmental outcomes after preeclampsia exposure alone [151, 152] or after grouped preeclampsia and gestational hypertension exposure [28] after adjustment for confounders. Schlapbach et al. (2010) [152] further demonstrated that postnatal complications of preterm birth including mechanical ventilation, bronchopulmonary dysplasia and sepsis had greater associations with poor neurodevelopment than the pathophysiological changes of preeclampsia exposure itself [152].

**Table 3:** Studies assessing the impact of preeclampsia exposure on infant neurodevelopment in infancy (birth – 2 years)

**First Author (Year)**	**Study Type**	**Exposure (Number)**	**Tool**	**Main Findings at 2 Years**^a^ **(PE versus NTP-exposed infants)**	**Adjusted Confounders**	**Comments**
Szymonowicz (1987) [23]	Case–control	PE (35) NTP (35)	BSID	MDI: PE lower PDI: ND	Nil	Cohort: preterm, VLBW infants^b^ ROB: Low
Spinillo (1994) [148]	Case–control	PE (68) NTP (184)	BSID	MDI: PE lower PDI: PE lower	Maternal age, SES, education	Cohort: preterm infants, PE group had expectant management ROB: Low
McCowan (2002) [153]	Prospective cohort	PE/GH (88) NTP (131)	BSID-II	MDI: PE/GH higher PDI: ND ND between < 32 weeks and > 32 weeks	Infant sex, GA, hospital stay, breastfeeding status, perinatal complications Maternal age, parity, ethnicity, smoking, education	Cohort: SGA infants^b^ Grouped PE and GH Assessed 18-month outcomes ROB: Low
Cheng (2004) [25]	Retrospective cohort	PE (25) NTP (54)	BSID-II	MDI: PE lower (mild delay from -1 to -2 SDs), ND (severe delay), ND between SGA PE and SGA NTP PDI: ND	Infant sex, GA, birthweight, lack of prenatal steroid, PPROM, intraventricular haemorrhage Maternal/paternal education, chronic lung disease	Cohort: VLBW, very preterm (< 32 weeks) infants^b^. Small sample ROB: Low
Silveria (2007) [26]	Prospective cohort	PE (40) NTP (46)	BSID-II	MDI: ND PDI: PE higher	Nil	Cohort: VLBW infants^b^ Small sample. Assessed 12, 18-month outcomes ROB: Low
Spinillo (2009) [154]	Prospective cohort	PE (185) NTP (596)	BSID-II	MDI: PE higher (female higher than male), SGA lower than non-SGA	Infant sex, GA, proportion of expected birthweight, SGA status, antenatal steroids, placental abruption, praevia, PPROM, non-reassuring fetal heart rate, chorioamnionitis, caesarean section, year of birth, umbilical artery pH = < 7.2 Maternal age, parity, education, SES, smoking	Cohort: preterm infants^b^ ROB: Low
Schlapbach (2010) [152]	Case–control	PE (33) NTP (33)	BSID-II	MDI: ND PDI: ND	Infant GA, birthweight, 2-year body weight, bronchopulmonary dysplasia, mechanical ventilation	Cohort: very preterm (< 32 weeks) infants^b^
Matić (2017) [28]	Retrospective cohort	PE/GH (261) NTP (1212)	Griffiths MDS, BSID-II	ND Long-term functional disability: SGA status, earlier GA and male sex were significant	Infant sex, GA, birthweight, surfactant therapy Maternal parity	Grouped PE and GH Cohort: infants aged 2–3 years, born very preterm (< 29 weeks). Powered to assess chronic lung disease, not just neurodevelopment ROB: Low
Degirmenci-oglu (2018) [155]	Retrospective cohort	PE (120) NTP (251)	BSID-II	MDI: PE higher PDI: ND Overall neurodevelopmental index: ND	Infant GA, birthweight, asphyxia, sepsis, intraventricular haemorrhage, necrotising enterocolitis Maternal hypothyroidism	Cohort: VLBW, very preterm (< 32 weeks) infants, but FGR infants were excluded^b^ Assessed 18- 24-month outcomes ROB: Low
Martikainen (1989) [24]	Prospective cohort	GH (14 preterm, 60 term) PE (31 preterm, 40 term) NTP (128 preterm, 175 term)	Denver	Term: PE/GH higher motor performance, visuo-auditory perception, and social abilities Preterm: PE lower fine motor and visuo-auditory perception, SGA lower than non-SGA	Infant sex, GA	Also assessed other HDPs. Cohort stratified by hypertension exposure, prematurity and SGA status^b^. Assessed 18-month outcomes ROB: Low
Gray (1998) [151]	Prospective cohort	GH (14) PE (79) NTP (107)	Griffiths-II, NSMDA	ND	Nil	Cohort: very preterm (24–32 weeks) infants^b^ ROB: Low
Johnson (2015) [149]	Prospective cohort	Preterm (638) Term (765)	PARCA-R	Preterm: PE was independent risk factor for cognitive impairment, preterm lower than term	Infant sex, SGA status Maternal ethnicity, SES	Cohort: late preterm infants (32–36 weeks)^b^ Assessed other perinatal variables, including PE ROB: Low
Wade (2016) [156]	Prospective cohort	HDP (23) NTP (478)	Many tools- see study	Social cognition: HDP lower	Infant age, sex, GA, birthweight Maternal age, gestational diabetes mellitus, thyroid problems, SES, smoking status	Grouped PE with other HDPs. Small sample Assessed 18-month outcomes ROB: Low
Warshafsky (2016) [80]	Prospective cohort	Mild PE (34) Severe PE (46) NTP (103)	ASQ	Severe PE was protective, higher GA reduced risk and FGR increased risk in both groups	Infant sex, GA, SGA status breastfeeding status, MgSO_4_ usage Maternal age, parity, ethnicity, smoking, SES, education	Cohort: FGR infants below 5^th^ centile Removed the mild PE subgroup due to poor numbers. Assessed 12, 24-month outcomes ROB: Low
Bharadwaj (2018) [157]	Case–control	PE (56) NTP (61)	DASII	Motor and mental development quotients: PE lower, maternal total antioxidant status was an independent motor development quotient predictor (PE group)	Infant GA, early onset sepsis, respiratory distress syndrome, necrotising enterocolitis Maternal total antioxidant status, Maternal and cord/baby protein carbonyl levels	No adjustment for SGA or prematurity status Assessed 12-month outcomes ROB: Low
Chen (2020) [158]	Prospective cohort	GH (233) PE (41) NTP (3669)	GDS	Social Behaviour Development Quotient: GH lower Neurodevelopmental delay: ND	Infant sex, GA, birthweight, mode of delivery, asphyxia neonatorum Maternal age, smoking, drinking, education, folic acid supplementation	Also studied chronic hypertension Assessed 6-month outcomes ROB: Low
Maher (2020) [159]	Prospective cohort	PE (709) NTP (10,425)	ASQ	ASQ failure: ND, ND between preterm vs term	Infant sex, SGA, prematurity Maternal age, ethnicity, BMI, gestational diabetes mellitus, education, SES	PE status determined by maternal recall. Assessed 9-month outcomes ROB: Low

*Abbreviations*: *ASQ* Ages and Stages Questionnaire [125], *BSID-II* Bayley Scales of Infant Development (2^nd^ edition) [138], *C-HTN*^†^ Complicated hypertension, *DASII* Developmental Assessment Scale for Indian Infants [160], *Denver* The Denver Developmental Screening Test [161], *FGR* Fetal growth restriction, *GA* gestational age at birth, *GDS* Gesell Developmental Schedules [162, 163], *GH* Gestational hypertension, *HDP* Hypertensive disorder of pregnancy, *HTN*, Hypertension, *MDI* Mean developmental index (BSID), *MDS* Griffiths Mental Development Scale [141], *ND* No difference, *NSMDA* Neurosensory Motor Developmental Assessment [164], *NTP* Normotensive pregnancy, *PARCA-R* Parent Report of Children’s Abilities- Revised [132], *PDI* Psychomotor development index (BSID), *PE* Preeclampsia, *PRROM* Preterm premature rupture of the membranes, *ROB* Risk of bias, *SES* Socioeconomic status, *SGA* Small for gestational age, *VLBW* Very low birth weight

^a^All results in the ‘Main Findings’ column are of infant developmental outcomes at 2 years, unless specified in the ‘Comments’ column

^b^Preterm birth was defined as birth < 37 weeks’ gestation. VLBW was defined as birthweight < 1500 g. SGA birth was defined as birthweight corrected for gestational age < 10^th^ centile. Study-specific definitions of ‘very preterm’ are specified in the ‘Comments’ column

When considering infant populations born not only preterm, but also of VLBW or SGA, studies assessing the impact of preeclampsia have reported similarly discrepant findings. Two small studies in VLBW, preterm infants, found those exposed to preeclampsia had lower BSID-II MDI scores at 2 years but no difference in PDI scores, suggesting preeclampsia exposure itself may contribute to poor mental development [23, 25]. However, Cheng et al. (2004) [25] found these differences were only associated with mild neurodevelopmental delay (-1 to -2 SDs from the mean) rather than severe delay (> -2 SDs), and found no differences when controlling for SGA status. In FGR infants, Warshafsky et al. (2016) [80] demonstrated that those exposed to severe preeclampsia were more likely to have failed at least one ASQ category at 12 and 24 months, especially the gross motor category, than those exposed to mild preeclampsia or normotensive pregnancies. This may reflect the clinical variability of mild versus severe disease. Similar to Martikainen et al. (1989) [24] however, they also reported that lower gestational age significantly contributed to the impact of severe preeclampsia, and FGR increased the risk in all groups, suggesting the impacts of preeclampsia on infant neurodevelopment may not be independent of these intermediary morbidities.

Alternatively, studies in these VLBW, SGA or preterm cohorts have suggested preeclampsia exposure may be neuroprotective and associated with a reduced risk of neurodevelopmental delay in one or more subcategories [26, 80, 153–155]. Two large cohort studies in preterm cohorts found preeclampsia-exposed infants had higher BSID-II MDI scores at 2 years [154, 155]. Although Spinillo (2009) [154] reported preeclampsia overall was associated with reduced risk of neurodisability, this finding may be explained by their normotensive preterm group being predominantly exposed to spontaneous birth or preterm premature rupture of the membranes, which carry increased risks of infection or inflammation that may be greater associated with abnormal neurodevelopment than preeclampsia exposure itself [154]. Furthermore, Spinillo (2009) [154] reported that although preeclampsia exerted a protective effect overall, the impaired neurodevelopment associated with male sex was higher for preeclampsia-exposed infants than their normotensive counterparts, suggesting a greater vulnerability of male infants to the pathophysiological changes of preeclampsia. Furthermore, McCowan et al. (2002) [153] and Silveira et al. (2007) [26] in SGA or VLBW cohorts, reported that preeclampsia-exposed infants had higher MDI and PDI scores respectively at 18 months. Although suggestive of a neuroprotective effect of preeclampsia, McCowan et al. (2002) [153] grouped preeclampsia and gestational hypertension exposure, and the other causes of SGA birth that normotensive controls were exposed to may mediate this finding, as they may be more strongly associated with neurodevelopmental delay than preeclampsia itself, similar to the complications of preterm birth, [153].

Few studies assessing infant neurodevelopment after preeclampsia exposure have been conducted in mixed cohorts including infants born at term or of an appropriate birthweight. As previously described, Martikainen et al. (1989) [24] found term preeclampsia-exposed infants had greater motor performance, visuo-auditory skills and social abilities at 18 months than both term normotensive, or preterm preeclampsia-exposed infants. In contrast, Wade (2016) [156] reported infants exposed to preeclampsia and other hypertensive pregnancy disorders had poorer social cognition at 18 months after full adjustment for confounders, however their study was retrospective in design, and limited by a small sample of preeclampsia-exposed infants. Similarly, Bharadwaj et al. (2018) [157] using a comparably validated foreign language tool to the BSID-II, reported preeclampsia exposure was independently associated with poorer motor and cognitive development at 1 year. They additionally reported that a lower maternal antioxidant status was an independent predictor of poorer motor development in the preeclampsia-exposed group, suggesting the intrauterine maternal oxidative stress present in preeclampsia may potentially contribute to impaired infant neurodevelopment. However, in larger cohorts, Chen et al. (2020) [158] and Maher (2020) [159] reported no difference in psychomotor developmental outcomes after full adjustment for perinatal confounders.

As such, while preeclampsia may not be associated with neuroprotective impacts in infancy, it remains inconclusive as to whether its underlying pathophysiological mechanisms negatively impact infant neurodevelopment independent of common perinatal confounders. Subsequently, further prospective studies with larger sample sizes, that include term infants born at an appropriate birthweight, and that use validated psychometric assessment tools such as the BSID-II, are indicated to disentangle the relationships of these variables. While these pathophysiological relationships remain unclear, clinically, preterm and SGA birth are common complications experienced by preeclampsia-exposed infants, and hence exposed infants may be at greater risk of neurodevelopmental impairment overall [7, 10, 57]. Although not the focus of this review, preeclampsia exposure may be additionally associated with other neurosensory disabilities including cerebral palsy [85, 165], blindness, deafness and intellectual disabilities [81, 165], which are often studied concurrently to psychomotor development and can assist in providing a greater understanding of the impact of preeclampsia on infant neurodevelopment as a whole.

## Limitations

The reviewed literature contains several limitations. Each study varied slightly in their definitions of preeclampsia, with most defining preeclampsia as new onset hypertension > 20 weeks gestation with varying degrees of proteinuria [18, 24–27, 29, 30, 32, 80, 148, 153–155, 158, 159], some using a broader definition of preeclampsia encompassing features of maternal end-organ dysfunction or uteroplacental insufficiency [23, 31, 151, 152, 157], one combining gestational hypertension and preeclampsia [28], and others poorly defining preeclampsia or relying on maternal report of preeclampsia during pregnancy [149, 156, 159]. Studies used a variety of local and international growth standards, including the WHO Growth Standards, to calculate z-scores for infant anthropometric measures which may not be appropriate for calculating the longitudinal growth outcomes of preterm populations. While studies were assessed as containing a low risk of bias based on methodological quality, the JBI tools do not consider sample size, and the aforementioned variances in study design, exposure definitions, growth assessments and control of confounders allow no definitive conclusions to be drawn, and we acknowledge that as a review article, our interpretation of the literature is subject to bias. However, our review aims to highlight trends in the literature and guide future study design rather than draw definitive conclusions regarding the impact of preeclampsia on child health.

## Conclusions

Preeclampsia is a serious pregnancy complication with significant consequences for both maternal and paediatric health. It is well established that preeclampsia causes FGR, SGA and preterm birth, and is associated with increased risk of cardiometabolic, neurodevelopmental and immunological morbidity in later life. Preeclampsia-exposed infants born SGA either do not demonstrate catch-up, especially those exposed to more severe or early-onset preeclampsia, or alternatively may experience rapid weight gain and catch-up growth, however perinatal confounders such as maternal BMI and postnatal feeding may influence this association. While most data suggest preeclampsia exposure may not impair infant motor and cognitive development independent of the influence of preterm and SGA birth, further research is required in larger cohorts born at term, controlling for perinatal confounders, and using standardised and validated assessment measures appropriate for individual child health and demographic characteristics, including gestational age at birth, SGA status, language, and neuropsychological disabilities. These may elucidate how the underlying pathophysiological mechanisms of preeclampsia impact infant health outcomes, and highlight the opportunity for early monitoring of infant growth and development before school age and the onset of later childhood morbidity. These may also indicate the need for novel therapeutic intervention, or early lifestyle intervention such as improving infant feeding practices, to optimise the future cardiometabolic and neurodevelopmental health outcomes of exposed infants. 

## Data Availability

No datasets generated or analysed for this review. All data reviewed is previously published. Figure created with BioRender.com.

## Abbreviations

ASQ: Ages and Stages Questionnaire
BMI: Body Mass Index
BSID-II/III: Bayley Scales of Infant Development (2^nd^ ed./3^rd^ Edition)
CVD: Cardiovascular disease
DOHaD: Developmental Origins of Health and Disease
FGR: Fetal growth restriction
JBI: Joanna Briggs Institute
MDI: Mental developmental index
PDI: Psychomotor developmental index
SD: Standard deviation
SGA: Small for gestational age
VLBW: Very low birthweight
WHO: World Health Organisation
